# Supplementary material for: Multi‐dataset identification of innovative feature genes and molecular mechanisms in keratoconus
Source: J Cell Mol Med. 2024 Sep 19;28(18):e70079. doi: 10.1111/jcmm.70079 (PMC11412914; doi:10.1111/jcmm.70079)
Supplement: Supplementary file 2 — Material S2. [file JCMM-28-e70079-s002.zip › 7.GSEA analysis/2.ATOH7/KEGG_CELL_ADHESION_MOLECULES_CAMS.html]

Details for gene set KEGG\_CELL\_ADHESION\_MOLECULES\_CAMS[GSEA]

|  || Dataset | ATOH7.group.cls#h\_versus\_l |
| Phenotype | group.cls#h\_versus\_l |
| Upregulated in class | h |
| GeneSet | KEGG\_CELL\_ADHESION\_MOLECULES\_CAMS |
| Enrichment Score (ES) | 0.41694808 |
| Normalized Enrichment Score (NES) | 1.3243985 |
| Nominal p-value | 0.073308274 |
| FDR q-value | 0.3310935 |
| FWER p-Value | 0.952 |
Table: GSEA Results Summary

  

Fig 1: Enrichment plot: KEGG\_CELL\_ADHESION\_MOLECULES\_CAMS      
 Profile of the Running ES Score & Positions of GeneSet Members on the Rank Ordered List

  

| SYMBOL | TITLE | RANK IN GENE LIST | RANK METRIC SCORE | RUNNING ES | CORE ENRICHMENT || 1 | JAM2 | na | 98 | 0.775 | 0.0337 | Yes |
| 2 | HLA-DQA1 | na | 299 | 0.625 | 0.0574 | Yes |
| 3 | CADM3 | na | 517 | 0.542 | 0.0767 | Yes |
| 4 | HLA-DPB1 | na | 612 | 0.518 | 0.0984 | Yes |
| 5 | NRXN3 | na | 728 | 0.493 | 0.1184 | Yes |
| 6 | JAM3 | na | 846 | 0.472 | 0.1372 | Yes |
| 7 | MADCAM1 | na | 934 | 0.458 | 0.1563 | Yes |
| 8 | CD40 | na | 1048 | 0.440 | 0.1738 | Yes |
| 9 | HLA-DQA2 | na | 1082 | 0.435 | 0.1934 | Yes |
| 10 | CDH2 | na | 1265 | 0.412 | 0.2076 | Yes |
| 11 | CLDN2 | na | 1390 | 0.398 | 0.2228 | Yes |
| 12 | NRCAM | na | 1430 | 0.393 | 0.2402 | Yes |
| 13 | CD274 | na | 1554 | 0.381 | 0.2546 | Yes |
| 14 | ESAM | na | 1601 | 0.377 | 0.2711 | Yes |
| 15 | NCAM1 | na | 1874 | 0.354 | 0.2799 | Yes |
| 16 | CDH4 | na | 1976 | 0.346 | 0.2933 | Yes |
| 17 | CLDN14 | na | 2270 | 0.325 | 0.3001 | Yes |
| 18 | ICAM1 | na | 2463 | 0.312 | 0.3093 | Yes |
| 19 | NRXN1 | na | 2491 | 0.310 | 0.3231 | Yes |
| 20 | PVR | na | 2760 | 0.295 | 0.3293 | Yes |
| 21 | SELL | na | 2849 | 0.288 | 0.3403 | Yes |
| 22 | CD40LG | na | 3305 | 0.265 | 0.3397 | Yes |
| 23 | HLA-DMB | na | 3372 | 0.261 | 0.3501 | Yes |
| 24 | ITGB8 | na | 3379 | 0.261 | 0.3622 | Yes |
| 25 | CD80 | na | 3860 | 0.242 | 0.3598 | Yes |
| 26 | HLA-DRA | na | 3924 | 0.239 | 0.3692 | Yes |
| 27 | HLA-DQB1 | na | 4600 | 0.215 | 0.3598 | Yes |
| 28 | HLA-DPA1 | na | 4808 | 0.207 | 0.3636 | Yes |
| 29 | CLDN6 | na | 5033 | 0.201 | 0.3666 | Yes |
| 30 | CLDN18 | na | 5084 | 0.198 | 0.3745 | Yes |
| 31 | PTPRM | na | 5367 | 0.189 | 0.3753 | Yes |
| 32 | SPN | na | 5641 | 0.181 | 0.3760 | Yes |
| 33 | CDH3 | na | 5817 | 0.176 | 0.3792 | Yes |
| 34 | CLDN17 | na | 5912 | 0.173 | 0.3847 | Yes |
| 35 | CNTNAP2 | na | 6217 | 0.165 | 0.3837 | Yes |
| 36 | CLDN10 | na | 6231 | 0.164 | 0.3910 | Yes |
| 37 | NEO1 | na | 6308 | 0.162 | 0.3965 | Yes |
| 38 | CD34 | na | 6379 | 0.160 | 0.4020 | Yes |
| 39 | CD6 | na | 6611 | 0.154 | 0.4025 | Yes |
| 40 | ITGA6 | na | 6683 | 0.152 | 0.4076 | Yes |
| 41 | CD226 | na | 6773 | 0.149 | 0.4121 | Yes |
| 42 | ITGAM | na | 6846 | 0.147 | 0.4169 | Yes |
| 43 | HLA-DRB4 | na | 7427 | 0.132 | 0.4064 | No |
| 44 | CD8B | na | 7450 | 0.132 | 0.4120 | No |
| 45 | VCAN | na | 7629 | 0.127 | 0.4129 | No |
| 46 | SDC3 | na | 8074 | 0.118 | 0.4056 | No |
| 47 | MAG | na | 8145 | 0.116 | 0.4090 | No |
| 48 | HLA-DRB5 | na | 8559 | 0.107 | 0.4021 | No |
| 49 | NFASC | na | 8692 | 0.104 | 0.4032 | No |
| 50 | CNTNAP1 | na | 8838 | 0.102 | 0.4039 | No |
| 51 | ITGAV | na | 8959 | 0.100 | 0.4051 | No |
| 52 | PECAM1 | na | 9243 | 0.095 | 0.4014 | No |
| 53 | HLA-DRB1 | na | 9300 | 0.094 | 0.4042 | No |
| 54 | NRXN2 | na | 9349 | 0.093 | 0.4072 | No |
| 55 | ITGB1 | na | 9539 | 0.090 | 0.4059 | No |
| 56 | CLDN15 | na | 10097 | 0.080 | 0.3936 | No |
| 57 | HLA-DOB | na | 10476 | 0.074 | 0.3862 | No |
| 58 | NCAM2 | na | 10696 | 0.071 | 0.3832 | No |
| 59 | CD276 | na | 11089 | 0.065 | 0.3749 | No |
| 60 | HLA-DRB3 | na | 11175 | 0.063 | 0.3754 | No |
| 61 | NLGN2 | na | 11439 | 0.060 | 0.3706 | No |
| 62 | GLG1 | na | 11935 | 0.053 | 0.3589 | No |
| 63 | VCAM1 | na | 12228 | 0.050 | 0.3528 | No |
| 64 | CDH5 | na | 12244 | 0.050 | 0.3547 | No |
| 65 | CD8A | na | 12662 | 0.044 | 0.3447 | No |
| 66 | CD58 | na | 12698 | 0.044 | 0.3457 | No |
| 67 | CLDN1 | na | 13367 | 0.037 | 0.3281 | No |
| 68 | HLA-G | na | 13698 | 0.033 | 0.3202 | No |
| 69 | HLA-DMA | na | 14540 | 0.025 | 0.2971 | No |
| 70 | HLA-A | na | 15242 | 0.019 | 0.2777 | No |
| 71 | HLA-C | na | 15354 | 0.018 | 0.2754 | No |
| 72 | HLA-DOA | na | 15373 | 0.018 | 0.2757 | No |
| 73 | CNTN2 | na | 15838 | 0.014 | 0.2629 | No |
| 74 | MPZ | na | 15903 | 0.014 | 0.2617 | No |
| 75 | CLDN16 | na | 15978 | 0.013 | 0.2602 | No |
| 76 | SDC4 | na | 16084 | 0.012 | 0.2577 | No |
| 77 | ICOSLG | na | 16605 | 0.008 | 0.2431 | No |
| 78 | MPZL1 | na | 17019 | 0.004 | 0.2314 | No |
| 79 | F11R | na | 17825 | -0.003 | 0.2082 | No |
| 80 | CADM1 | na | 18168 | -0.005 | 0.1986 | No |
| 81 | HLA-E | na | 18198 | -0.006 | 0.1980 | No |
| 82 | PDCD1 | na | 18241 | -0.006 | 0.1971 | No |
| 83 | ICOS | na | 18396 | -0.008 | 0.1930 | No |
| 84 | CLDN9 | na | 18876 | -0.012 | 0.1797 | No |
| 85 | SDC1 | na | 19019 | -0.013 | 0.1762 | No |
| 86 | SELPLG | na | 19236 | -0.015 | 0.1707 | No |
| 87 | ITGA8 | na | 19267 | -0.015 | 0.1705 | No |
| 88 | HLA-F | na | 19312 | -0.016 | 0.1700 | No |
| 89 | NEGR1 | na | 19447 | -0.017 | 0.1669 | No |
| 90 | CD99 | na | 19501 | -0.017 | 0.1662 | No |
| 91 | CDH1 | na | 19700 | -0.019 | 0.1613 | No |
| 92 | ITGB2 | na | 20232 | -0.024 | 0.1471 | No |
| 93 | SELE | na | 20266 | -0.024 | 0.1473 | No |
| 94 | CLDN4 | na | 21632 | -0.039 | 0.1097 | No |
| 95 | PTPRF | na | 21634 | -0.039 | 0.1115 | No |
| 96 | NLGN4X | na | 22003 | -0.043 | 0.1029 | No |
| 97 | CLDN19 | na | 22264 | -0.046 | 0.0975 | No |
| 98 | PDCD1LG2 | na | 22348 | -0.047 | 0.0973 | No |
| 99 | ICAM2 | na | 22386 | -0.047 | 0.0985 | No |
| 100 | CLDN8 | na | 22405 | -0.047 | 0.1002 | No |
| 101 | L1CAM | na | 22419 | -0.047 | 0.1021 | No |
| 102 | NLGN1 | na | 22744 | -0.051 | 0.0951 | No |
| 103 | ITGA9 | na | 23027 | -0.055 | 0.0896 | No |
| 104 | NLGN3 | na | 23055 | -0.056 | 0.0914 | No |
| 105 | HLA-B | na | 23071 | -0.056 | 0.0936 | No |
| 106 | CD4 | na | 23285 | -0.059 | 0.0902 | No |
| 107 | CNTN1 | na | 23304 | -0.059 | 0.0925 | No |
| 108 | CLDN5 | na | 23339 | -0.059 | 0.0943 | No |
| 109 | ALCAM | na | 23826 | -0.067 | 0.0834 | No |
| 110 | CLDN11 | na | 23906 | -0.068 | 0.0844 | No |
| 111 | CD28 | na | 24062 | -0.071 | 0.0832 | No |
| 112 | CD2 | na | 24294 | -0.074 | 0.0800 | No |
| 113 | ICAM3 | na | 24336 | -0.075 | 0.0823 | No |
| 114 | SIGLEC1 | na | 25172 | -0.088 | 0.0624 | No |
| 115 | ITGA4 | na | 26362 | -0.112 | 0.0333 | No |
| 116 | CLDN7 | na | 26468 | -0.114 | 0.0356 | No |
| 117 | ITGB7 | na | 26886 | -0.123 | 0.0294 | No |
| 118 | CLDN3 | na | 27052 | -0.127 | 0.0306 | No |
| 119 | ITGAL | na | 27827 | -0.146 | 0.0151 | No |
| 120 | OCLN | na | 27893 | -0.148 | 0.0202 | No |
| 121 | SELP | na | 28358 | -0.161 | 0.0144 | No |
| 122 | PTPRC | na | 28489 | -0.164 | 0.0184 | No |
| 123 | CDH15 | na | 29560 | -0.197 | -0.0033 | No |
| 124 | SDC2 | na | 29806 | -0.204 | -0.0007 | No |
| 125 | CLDN23 | na | 30304 | -0.222 | -0.0046 | No |
| 126 | CD22 | na | 31362 | -0.262 | -0.0228 | No |
| 127 | CLDN20 | na | 33117 | -0.377 | -0.0558 | No |
| 128 | CD86 | na | 33693 | -0.439 | -0.0517 | No |
| 129 | CTLA4 | na | 33922 | -0.481 | -0.0356 | No |
| 130 | CLDN22 | na | 34724 | -1.248 | 0.0001 | No |
Table: GSEA details [plain text format]

  

Fig 2: KEGG\_CELL\_ADHESION\_MOLECULES\_CAMS      
 Blue-Pink O' Gram in the Space of the Analyzed GeneSet

  

Fig 3: KEGG\_CELL\_ADHESION\_MOLECULES\_CAMS: Random ES distribution      
 Gene set null distribution of ES for **KEGG\_CELL\_ADHESION\_MOLECULES\_CAMS**
